# Age-dependent vascular and neurological characteristics of CADASIL are recapitulated in Notch3 mutant zebrafish, implicating a role for type IV collagen in disease progression

**DOI:** 10.1186/s40478-026-02333-8

**Published:** 2026-06-03

**Authors:** Tohgo Kanoh, Shiho Oubayashi, Kengo Furukawa, Kosuke Fujimoto, Amane Inoue, Takamasa Mizoguchi, Masashi Yamaguchi, Azusa Takahashi-Nakaguchi, Yoshinori Hasegawa, Osamu Ohara, Ichio Aoki, Motoyuki Itoh

**Affiliations:** 1 Graduate School of Pharmaceutical Sciences, Chiba University, 1-8-1 Inohana, Chuo-Ku, Chiba, 260-8675 Japan; 2 Medical Mycology Research Center, Chiba University, 1-8-1 Inohana, Chuo-Ku, Chiba, 260-8673 Japan; 3 Department of Applied Genomics, Kazusa DNA Research Institute, 2-6-7 Kazusa Kamatari, Kisarazu, Chiba 292-0818 Japan; 4 Institute for Quantum Medical Science, National Institutes for Quantum Science and Technology, 4-9-1 Anagawa, Inage-Ku, Chiba, 263-8555 Japan; 5 Research Institute of Disaster Medicine, Chiba University, 1-8-1 Inohana, Chuo-Ku, Chiba, 260-8675 Japan; 6 Health and Disease Omics Center, Chiba University, 1-8-1 Inohana, Chuo-Ku, Chiba, 260-8675 Japan

**Keywords:** CADASIL, Notch3, Cerebral small vessel disease, Vascular dementia, Collagen, Serpinh1, Zebrafish

## Abstract

CADASIL is a hereditary cerebral small vessel disease caused by *NOTCH3* mutations, leading to age-dependent vascular and neurological impairments. Despite its clinical impact, the underlying pathogenic mechanisms remain poorly understood, partly because existing murine models often fail to fully replicate the broad pathological spectrum observed in humans. Here, we established and characterized a novel zebrafish (Danio rerio) model carrying the Notch3 p.C680S mutation. Our mutant zebrafish mimicked key age-related CADASIL features, including cerebrovascular dysfunction, telencephalic atrophy, cognitive impairment, and the deposition of granular osmiophilic material (GOM). Integrated transcriptomic and proteomic analyses revealed a significant downregulation of type IV collagen-related molecules, including *serpinh1b*, *col4a1*, and *col4a2.* These molecular findings were corroborated by immunostaining, which confirmed reduced perivascular accumulation of type IV collagen around cerebral blood vessels. Furthermore, AlphaFold 3-based structural modeling categorized the p.C680S variant as a “nonconventional” NOTCH3 mutation. These findings validate the zebrafish as a valuable vertebrate model for CADASIL and raise the possibility that diminished type IV collagen levels contribute to CADASIL pathogenesis, particularly in cases of nonconventional NOTCH3 mutations.

**Supplementary Information:**

The online version contains supplementary material available at 10.1186/s40478-026-02333-8.

## Introduction

Cerebral autosomal dominant arteriopathy with subcortical infarct and leukoencephalopathy (CADASIL) is the most frequent age-related cerebral small vessel disease (CSVD), leading to ischemic stroke and vascular dementia [9, 31]. The worldwide prevalence of CADASIL is approximately estimated to be 3.4 per 1000 individuals [63]. Despite its significant clinical burden, no effective therapeutic strategies are currently available.

CADASIL is primarily caused by point mutations in the *NOTCH3* gene, which plays a crucial role in maintaining vascular wall integrity in adulthood [31, 82]. In line with this, numerous animal models harboring CADASIL-associated NOTCH3 mutations have been developed. Several transgenic mouse models carrying specific NOTCH3 mutations (e.g., p.R182C, p.R169C, p.R90C, or p.R75Q) have recapitulated key pathological features, including vascular abnormalities and the deposition of granular osmiophilic material (GOM)—abnormal protein aggregations containing the NOTCH3 extracellular domain that serve as a diagnostic marker [13, 21, 33, 61, 64]. However, these models frequently fail to fully recapitulate the human disease spectrum; for instance, they typically exhibit no or mild cognitive impairment, and often lack other key pathological features, such as significant white matter lesions [15, 21, 23, 46]. In addition, none of the knock-in models carrying NOTCH3 mutation, such as p.R170C or p.R142C, have demonstrated the full spectrum of CADASIL pathology [47, 81]. These limitations underscore the need for complementary animal models that faithfully recapitulate cognitive impairment, thereby facilitating elucidation of the underlying pathogenic mechanisms.

A recent study proposed a classification dividing CADASIL into two types—”conventional” and “nonconventional”—based on pathological features and NOTCH3 mutant structure [27]. Pathologically, the conventional type is more frequent in temporopolar lesions, while the nonconventional type is linked with pro-hemorrhagic CADASIL [27]. The conventional NOTCH3 mutations are typically cysteine-altering variants that create solvent-exposed cysteine residues (e.g., p.R182C, p.R169C, and p.R90C) [27]. Conversely, the nonconventional NOTCH3 mutations are either cysteine-sparing variants or cysteine-altering variants that do not result in solvent-exposed cysteine residues (e.g., p.R75P/Q and p.C606R) [27]. These findings indicate that distinct pathomechanisms underlie CADASIL, depending on whether the NOTCH3 mutation is conventional or nonconventional. Therefore, it is essential to utilize animal models carrying the same type of NOTCH3 mutation to accurately recapitulate the pathologies of CADASIL. In patients in East Asia (especially in Japan and Korea), NOTCH3 p.R75P, classified as a nonconventional mutation, is one of the most frequent mutations [7, 27, 78]. However, only one transgenic mouse model carrying a nonconventional mutant NOTCH3 (p.R75Q) has been reported [21], and it has been shown to exhibit no detectable abnormalities in the cerebral white matter.

In recent years, zebrafish have emerged as a promising model for age-related diseases, as their cerebral blood flow and brain clearance systems more closely parallel human physiology than those of murine models [29, 35, 50]. For example, the zebrafish model of Alzheimer’s disease with a knockout of *Psen1* exhibited anxiety-like behavior in adulthood, which was not recapitulated in the mouse model [65, 70]. Moreover, a zebrafish model of Fabry disease, a form of CSVD, closely replicates the human pathologies [16]. These studies suggest that zebrafish hold significant potential for recapitulating CADASIL pathologies, potentially addressing phenotypes that are difficult to model in mice. Despite this promise, a zebrafish model of CADASIL has not yet been established.

Therefore, this study aims to investigate whether a zebrafish model of CADASIL can mimic the pathological features observed in human patients and to elucidate the mechanisms underlying its progression. Our findings demonstrate that this zebrafish model successfully recapitulates key CADASIL features, including cognitive impairment. Moreover, based on its structural attributes, the mutation carried by this model appears to correspond to the nonconventional type. In addition, the zebrafish model provides a framework for proposing a potential mechanism underlying CADASIL progression, especially in cases involving nonconventional mutations.

## Materials and methods

### Zebrafish

The *notch3*^*fh334*^ line was originally generated as part of large-scale TILLING mutagenesis screen and obtained from ZIRC [53]. The *nacre* line was a gift from Dr. Zon (Howard Hughes Medical Institute) [85]. The *Tg(gata1:mRFP)*^*ko05*^ line was a gift from Dr. Kawahara (University of Yamanashi) [38]. The *Tg(kdrl:EGFP)*^*s843*^ line was obtained from ZIRC [6]. The *TgBAC(abcc9:gal4ff)* line was a gift from Dr. Mochizuki (National Cerebral and Cardiovascular Center Research Institute) [80]. The *Tg(UAS:EGFP)* line was a gift from Dr. Kawakami (National Institute of Genetics) [2]. The zebrafish were raised and maintained according to standard conditions [84] with approval by the Institutional Animal Care and Use Committee at Chiba University (nos 1–170, 2–174, 3–62, 4–6, 5–70, 6–86, 7–22, 30–442). Both males and females were used in all experiments. Wild-type (WT) siblings lacking the mutant allele were used as controls for all experiments. The dissection was performed between 3 p.m. and 6 p.m.

### Live imaging and measurement of perfused cerebral vessel fraction

The live imaging and measurement of perfused cerebral vessels were performed as previously described [50]. First, zebrafish were anaesthetized by diluting ethylene glycol monophenyl ether (Wako) with breeding water. After anesthesia, the fish were fixed by sandwiching it in the notch of the sponge. A peristaltic pump was used to supply 0.04% anesthetic to the mouth of the fish at a flow rate of 5 ml/min. Imaging was performed by confocal microscopy (Leica SP8) with a 10 × water immersion lens. After imaging, the breeding water was recirculated using a peristaltic pump, and once the fish had recovered from anesthesia, they were returned to the breeding system.

For analyzing the perfused cerebral vessel fraction, the same individuals of *Tg(kdrl:EGFP); Tg(gata1:mRFP)* were analyzed monthly starting at 7 months of age. Images taken by confocal microscopy were processed by Fiji (NIH). After creating Z-projection images, we manually measured the length of blood vessels with blood flow and without blood flow, respectively. The perfused cerebral vessel fraction was evaluated as the percentage of perfused blood vessel length relative to the total vessel length.

### Imaging and quantification of caudal fin blood accumulation

The zebrafish was anesthetized by diluting ethylene glycol monophenyl ether (Wako) with breeding water. The observation regions for blood accumulations were selected with reference to a previous study [92]. The caudal fin and caudal fin base images were captured by a stereomicroscope (Leica MZ16). Ratio of blood accumulation indicates the proportion of individuals exhibiting blood in the region of interest to the total number of observed zebrafish.

### Measurement of learning ability

Learning ability was assessed using an active avoidance test with discrete spaced training, as previously reported [50, 90]. The experimental tank was divided into two sections by a central partition through which fish could freely move through the gap below and was filled to a depth of 5 cm using fish water. The zebrafish were transferred to the experimental tank and given 10 min for adaptation to the new environment. After the adaptation time, a trial was initiated. The side with the zebrafish was designated as the shock-side; the shock side was illuminated with green light as the conditioned stimulus. After CS for 12 s, an electric shock was delivered for a maximum of 12 s as an unconditioned stimulus if the zebrafish were on the green side (shock-side), while if the zebrafish had moved to the white side (no-shock-side), the electric shock was inactivated promptly. This electric shock was set to stop when the fish fled to the no-shock-side. In successive spaced training, the zebrafish was given a one-hour quiescent period after 25 trials, for a total of 50 trials. The avoidance rate, defined as the percentage of instances in which the zebrafish escaped to the no-shock-side in response only to green light presentation, was used as an index of learning and memory ability.

### Free movement pattern (FMP) Y-maze test

Working memory was assessed using free movement pattern (FMP) Y-maze test. The Y-maze tank was prepared using 3D printer and designed based on a previous study [10]. The dimensions of the Y-maze tank were as follows: L50, W20, H140 (mm).

The maze floor was illuminated by an LED panel, and 6 maze tanks were simultaneously put on the LED panel. After the maze tanks were filled with 300 mL of aquarium water, the fish were put into the tank and allowed to freely swim for 60 min. Their behavior was recorded by a digital camera (EX-F1, Casio) positioned above the LED panel. Positional data were extracted from the video with idtrackerai [60], and tetragrams (four choices of left/right combinations) were calculated with R. The alternation rate, defined as the percentage of alternations (LRLR or RLRL) divided by the total tetragram, was used as an index of working memory.

### Electron microscopy

Brain tissues were fixed overnight at 4 °C in 2.5% glutaraldehyde and 2% paraformaldehyde prepared in 0.1 M phosphate buffer (pH 7.4). The tissues were post-fixed with 1% osmium tetroxide for 60 min at 4 °C, dehydrated through a graded ethanol series, and embedded in Quetol 812 (Nisshin EM Co., Ltd., Tokyo, Japan). Semi-thin Sects. (0.5 µm thick) were cut, mounted on glass slides, stained with toluidine blue, and examined by light microscopy to confirm the presence of blood vessels. Ultrathin Sects. (70 nm) were cut with a diamond knife (SYM knife, SYMTEK Co., Ltd., Tokyo, Japan) using an Ultracut S ultramicrotome (Leica, Switzerland), collected on 2 × 1 mm single-slot grids, and gently placed on Formvar support films [87]. The sections were double-stained with uranyl acetate and lead citrate and observed using a JEM-1400 transmission electron microscope (JEOL Ltd., Tokyo, Japan) operated at 60 kV [88].

### Analysis of the number of pericytes

The imaging method and area are the same as those used in perfused cerebral vessel fraction analysis. The number of pericytes was manually counted by using Fiji. Imaging was performed monthly in the same individuals. The relative number of pericytes was calculated as a percentage of the number present at 6 months of age.

### RNA isolation, cDNA synthesis, and qRT-PCR analysis

Total RNA of the zebrafish whole brain was extracted and purified by Sepasol-RNA I Super G (Nacalai Tesque) according to the manufacturer’s instructions. cDNA synthesis was conducted using ReverTra Ace (Toyobo). The mRNA expression levels were quantified by qRT-PCR with THUNDERBIRD SYBR qPCR Mix (Toyobo) on LightCycler 96 System (Roche). Expression levels were normalized against those of *rpl13a*, and the relative values of each data point were calculated using the ΔΔCt method.

Primer sequences are as follows:

- *abcc9* (forward), 5’- TGGGCAACAGCGCATTGATAGG -3’.
- *abcc9* (reverse), 5’- ATGTTCTCCCACGCATAGAGCTTC -3’.
- *pdgfrß* (forward), 5’- AGCCAGCATCAACATCACTG -3’.
- *pdgfrß* (reverse), 5’- TTCAGCTCCACGTTTTCTCC -3’.
- *serpinh1b* (forward), 5’- CCGCCAAGACAACTGATGGAAAG -3’.
- *serpinh1b* (reverse), 5’- TCTTGTGGTGGAACTTCTCATCCC -3’.
- *col4a1* (forward), 5’-ATGGCTGGGCTTCACTCTGGATTG -3’.
- *col4a1* (reverse), 5’- TTCAAGACAAGAGCCAGGAGAAGCC -3’.
- *col4a2* (forward), 5’- CGGTATGACGGCCTATCACT -3’.
- *col4a2* (reverse), 5’- TAAACGGCCTCCACAGAGTT -3’.

### MRI measurements

Zebrafish were euthanized and soaked in 2 mM Gd-DTPA contrast agent (Gadopentetate dimeglumine, Magnevist, Bayer) for one week at 4 °C. Then, zebrafish were inserted into a plastic sample tube and immersed in a fluorine compound (Fomblin, CF_3_O[-CF(CF_3_)CF_2_O-]_x_(-CF_2_O-)_y_CF_3_, Solvay Solexis, NJ, USA) to remove signal contamination from the surrounding water or air. The sample tube was set on the animal cradle to obtain the MRI measurements. Preclinical horizontal MRI scanner (7-T, 20 cm bore size, Biospec Avance-IV, Bruker Biospin, Ettlingen, Germany) with cryogenic 2-ch phased array coil for transmission and reception (Bruker Biospin) was used. The software for the MRI data acquisition and reconstitution used were the ParaVision 5.1 (Bruker Biospin). Following standard frequency adjustment routines, pilot scans (tripilot sequence) were used to accurately position the animal head inside the magnet.

For high resolution 3D anatomical MR imaging, 3D FLASH (fast low-angle shot) sequences were used with the following parameters: repetition time = 50 ms, echo time = 7 ms, flip angle = 60 degree, field of view = 4.8 × 7.0 × 4.25 mm^3^, matrix size = 192 × 280 × 170, spatial resolution = 25 × 25 × 25 µm^3^, fat suppression = off, and number of averages = 16. The acquisition time was approximately 10.58 h. The cerebral volumes were measured using OsiriX Lite software (Pixmeo SARL, Bernex, Switzerland), and the representative example of volume measurement is shown in Supplementary Fig. 1.

### RNA-seq analysis

Total RNA of the zebrafish whole brain was extracted and purified by Sepasol-RNA I Super G. RNA-seq library was prepared using SureSelect Strand-Specific RNA Library Prep System (Agilent Technology). The RNA-seq library was sequenced on a HiSeq 2500 system (Illumina), performing 100 bp paired-end reads, and we obtained > 40 million reads for each sample. The reads were aligned to the zebrafish reference genome (GRCz11) using CLC genomics workbench (QIAGEN, Hilden, Germany). CPM quantification and differentially expressed genes analysis were performed by the CLC Genomics Workbench. The zebrafish gene IDs of differentially expressed genes were converted to human gene IDs using g:Profiler [40], and the resulting human homolog genes were subjected to an enrichment analysis with Metascape [94]. Principal component analysis (PCA) was conducted on the top 1000 most variable genes, as determined by variance across samples, using the stats package in R [74]. The results were visualized with the factoextra package [36].

### Protein sample preparation for LC–MS/MS

After adding three volumes of ethanol to the organic phase of Sepasol-RNA I Super G and incubating at room temperature for 15 min, total proteins were precipitated by centrifugation at 15,000 g for 15 min at 4 °C. The pellet was resuspended in 100 mM Tris–HCl (pH 8.0) containing 4% SDS, 20 mM NaCl, and 10% ACN, and solubilized using an ultrasonic homogenizer. Protein concentration was determined using the BCA assay and adjusted to 0.15 µg/µl using the same buffer. Protein clean-up was performed based on the SP3 method [24, 25] with a 1:1 mixture of hydrophilic and hydrophobic Sera-Mag SpeedBead Carboxylate-Modified Magnetic Particles (Cytiva). Proteins were digested with Trypsin/Lys-C mix (Promega) at 37 °C for 14 h. Then, disulfide bonds were reduced and alkylated by adding 10 mM TCEP and 40 mM 2-chloroacetamide at 80 °C for 15 min. Digested peptides were acidified with 5% TFA (trifluoroacetic acid) and desalted using reversed-phase spin columns (GL-Tip SDB, GL Sciences). Peptides were resuspended in 0.1% TFA containing 0.01% DMNG as described in a previous report [41], and quantified using Fluorometric Peptide Assay (Cat# 23,290, Thermo Scientific). Samples were adjusted to a final concentration of 200 ng/µl for nanoLC-MS/MS analysis.

### NanoLC-MS/MS

The nanoLC-MS/MS analysis was performed by an Orbitrap Exploris 480 MS (Thermo Fisher Scientific) equipped with an UltiMate 3000 RSLCnano LC System (Thermo Fisher Scientific). The digested peptides were injected onto a 30 cm C18 column (ID 75 µm, 1.7 µm, 100 Å, CoAnn Technologies) at 50 °C and then separated with a 100-min gradient (A = 0.1% formic acid in water, B = 0.1% formic acid in 80% ACN) consisting of 7% B at 0 min, 37% B at, and 70% B from 94 to 100 min at a flow rate of 150 nL/min. The peptides eluted from the column were analyzed by DIA-MS. MS1 spectra were collected in the range of *m/z* 495 to 745 at a 60,000 resolution to a set an AGC target 3 × 10^6^ and a maximum injection time of “Auto”. MS2 spectra were collected at *m/z* 200 to 1,800 at a 45,000 resolution to set an AGC targets of 3 × 10^6^, a maximum injection time of “Auto”, and stepped normalized collision energies of 26%. The isolation width for MS2 was configured at 4 m*/z*, and for the 500 to 740 m*/z* window pattern, an optimized window arrangement was utilized in Scaffold DIA.

#### DIA-MS data analysis

The DIA-MS data were queried against the in silico zebrafish spectral library, using DIA-NN 1.8.1 [11]. The spectral library was generated from the Danio-rerio (Zebrafish) (Brachydanio-rerio) UniProtKB/Swiss-Prot database (proteome ID UP000000437, 26,166 entries, downloaded on December 15, 2023) using DIA-NN. The parameters for generating the spectral library were as follows: digestion enzyme, trypsin; missed cleavages, 1; peptide length range, 7–45; precursor charge range, 2–4; precursor m/z range 490–750; fragment ion *m/z* range, 200–1,800. Additionally, “FASTA digest for library-free search/library generation,” “deep learning-based spectra, RTs and IM prediction,” and “n-term M excision” were enabled. For the DIA-NN search, the following parameters were applied: a mass accuracy of 10 ppm, MS1 accuracy of 10 ppm, protein inference based on genes, utilization of neural network classifiers in single-pass mode, quantification strategy using Robust LC (high precision), and cross-run normalization set to “RT-dependent.” Furthermore, “Unrelated runs,” “Use isotopologues,” “Heuristic protein inference,” and “No shared spectra” were enabled. The threshold for protein identification was set to 1% or less for both precursor and protein false discovery rates. Protein quantification values were aggregated over the quantification values of unique peptides as calculated by DIA-NN. The enrichment analysis and PCA of differentially expressed proteins were performed by the same method as RNA-seq analysis.

### Brain Sectioning for Immunofluorescence

The dissected brain was fixed with 4% PFA in PBS overnight. For immunohistochemistry, the fixed brain was embedded in 2.5% agarose gel in PBS and then sectioned at a thickness of 100 µm by Neo-LinearSlicer (DOSAKA EM).

### Immunofluorescence

For collagen staining, 100-µm-thick sections of the telencephalon were prepared. Sections were cleared by incubation in PBST (1 × PBS containing 0.3% Triton X-100) for 1 h at room temperature. Antigen retrieval was performed by incubating sections in 6 M urea and 0.1 M glycine (pH 3.5) for 15 min. Blocking was conducted by incubating sections in 1% BlockAce (DS Pharma Biomedical) in PBST for 1 h. Primary antibodies (monoclonal anti-type IV collagen JK2 [68] (gift from Dr. Matsuyama in Shigei Medical Research Institute), 1:500; anti-GFP pAb (598, MBL), 1:500) were applied in PBST and incubated for 24 h at 4 °C. After six washes in PBST, sections were incubated with secondary antibodies (Alexa Fluor 552 AffiniPure Donkey anti-rabbit IgG (H + L) (711–545-152, Jackson Immuno Research) and Alexa Fluor 594 AffiniPure Donkey anti-rat IgG (H + L) (712–585-153, Jackson Immuno Research), all 1:500) overnight at 4 °C. Following five PBST washes and a final rinse in PBS, sections were mounted using Fluoromount (DIAGNOS).

For the assessment of collagen accumulation, the vascular perimeter was initially measured. As type IV collagen is not present within the vascular lumen, collagen signals detected within the lumen were regarded as background and excluded. The positive signal for type IV collagen was binarized using the automatic local threshold “Midgrey” method in Fiji, and the area of type IV collagen was calculated. The collagen accumulation level was calculated by the Area/Perimeter ratio.

### Structural analysis of the Notch3 protein

The structure of Notch3 EGFr 18 domain, including p.C680S mutation, was predicted using AlphaFold 3 (AF3 Webserver, https://alphafoldserver.com/) [1].

### Statistical analysis

Statistical significance was determined by appropriate tests as shown in the figure legend using GraphPad Prism (GraphPad software).

## Results

### *Notch3*^*fh334/*+^ zebrafish show cerebrovascular pathologies dependent on aging

**Fig. 1:**
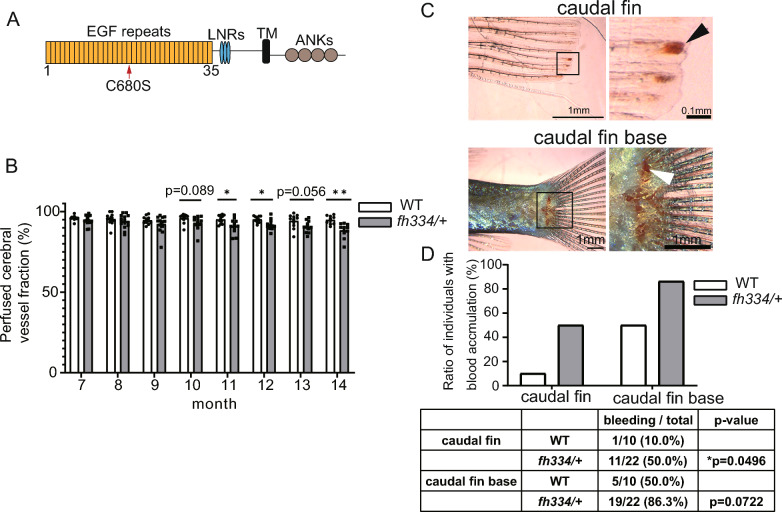
Age-dependent vascular dysfunction in *notch3*^*fh334/*+^ zebrafish. **A** Schematic diagram showing the notch3 *fh334* mutation (p.C680S). **B** Longitudinal analysis of the perfused cerebral vessel fraction in the same individual fish at monthly intervals. Numbers of analyzed WT fish were 12 (7 months), 12 (8 months), 11 (9 months), 11 (10 months), 10 (11 months), 10 (12 months), 10 (13 months), and 9 (14 months). Numbers of analyzed *notch3*^*fh334/*+^ fish were 14 (7 months), 14 (8 months), 13 (9 months), 11 (10 months), 11 (11 months), 11 (12 months), 11 (13 months), 10 (14 months). **C** Representative images of blood accumulation in the caudal fin and caudal fin base. Arrowheads indicate sites of the accumulation of blood. Scale bars are as indicated. **D** Proportion of individual zebrafish exhibiting blood accumulation in the caudal fin or its base. Numbers of analyzed fish are 10 (WT) and 22 (*notch3*^*fh334/*+^). Error bars in **B** represent the mean ± SEM, with each dot indicating an individual value. *p < 0.05 and **p < 0.01 by unpaired two-tailed *t*-test Data in D were analyzed using Fisher’s exact test

To investigate CADASIL-associated pathology and pathomechanisms, we used *notch3*^*fh334*^ zebrafish, which harbor a CADASIL-like mutation that substitutes the 680th cysteine with serine (p.C680S) (Fig. 1A) [53]. This position (p.C680S) in zebrafish corresponds to 704th cysteine in human NOTCH3, which has been reported to be potentially pathogenic [54]. The amino acid sequence identity between human and zebrafish Notch3 is 54%, and previous studies have reported that their functions and expression patterns are largely conserved [83, 92]. We used *notch3*^*fh334/*+^ (heterozygous) zebrafish because the majority of human CADASIL patients are heterozygous, and the symptoms in individuals with homozygous mutations are reported to resemble those of heterozygous patients [52].

Cerebral perfusion, defined as the volume of blood flowing through a given volume of cerebral tissue per unit time, is reported to decline in the early stages of CADASIL progression [37, 77, 79]. Therefore, a reduction in cerebral perfusion is considered an early symptom that precedes other manifestations of CADASIL. Since real-time measurement of the volume of blood flow in living zebrafish is technically challenging, we calculated the perfused vessel fraction as the proportion of vessels retaining blood flow (relative to total vessel length) using snapshot images. To analyze the perfused vessel fraction in *notch3*^*fh334/*+^ zebrafish and determine the onset of this reduction, we performed long-term live imaging using *notch3*^*fh334/*+^ zebrafish crossed with *Tg(kdrl:EGFP)*^*s843*^*;Tg(gata1:mRFP)*^*ko06*^*;nacre* zebrafish, in which vascular endothelial cells and erythrocytes are fluorescently labeled. We captured the images of the telencephalon which includes a region homologous to the mammalian cortex and hippocampus [19]. Live-imaging analysis of perfused cerebral vessel fraction revealed an age-dependent decline in both wild-type (WT) and *notch3*^*fh334/*+^ zebrafish (Fig. 1B). Although the perfused cerebral vessel fraction was not significantly different between the two groups until 10 months of age, *notch3*^*fh334/*+^ zebrafish showed a significant reduction in perfused cerebral vessel fraction compared to WT from 11 months of age (Fig. 1B). To further investigate the vascular impairment, we examined whether peripheral vessels are compromised by CADASIL mutations, as pathogenic Notch3 variants are known to affect small vessels outside the brain [55, 56]. We analyzed peripheral vessel abnormalities by evaluating the accumulation of blood within the caudal fin and its base, as described in a previous study [92] (Fig. 1C; Supplementary Fig. 2). The results showed that *notch3*^*fh334/*+^ zebrafish exhibited an accumulation of blood within the caudal fin and its base (Fig. 1D), suggesting that peripheral vessel function may also be compromised in *notch3*^*fh334/*+^ zebrafish. Although less frequently reported, peripheral arterial dysfunction and accumulation of blood in peripheral tissues such as skin and retina have been noted in a CADASIL patient and in a mouse model [8, 56, 58, 59].

Together, these data suggest that *notch3*^*fh334/*+^ zebrafish develop age-dependent vascular dysfunction mimicking human CADASIL.

### Age‑dependent memory impairment in *notch3*^***fh334/***+^ zebrafish

**Fig. 2:**
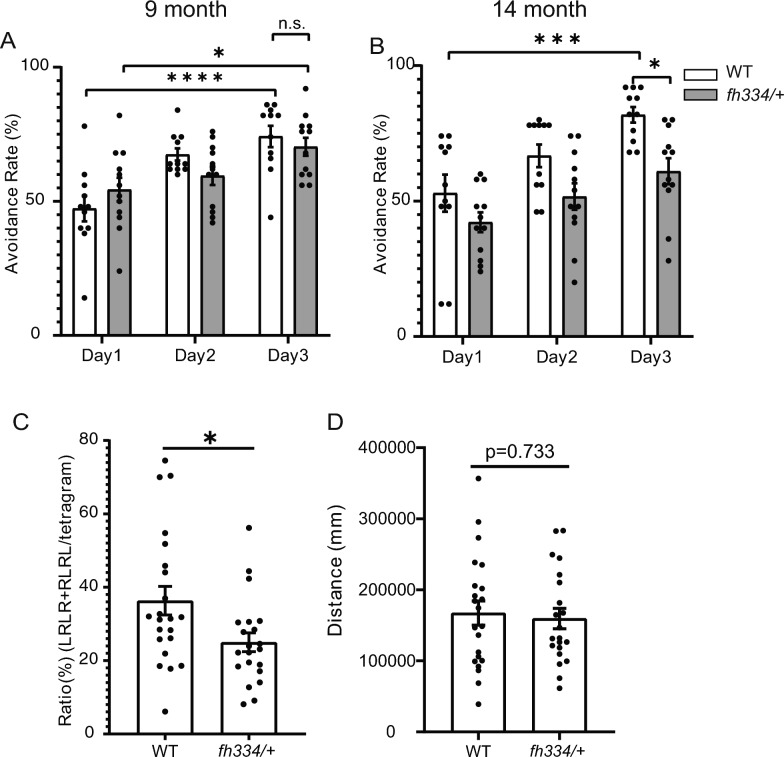
Impairment of learning ability and working memory in *notch3*^*fh334/*+^ zebrafish. **A** Temporal changes in active avoidance performance at 9 months of age. Numbers of analyzed fish are 11 (WT) and 12 (*notch3*^*fh334/*+^). **B** Temporal changes in active avoidance performance at 14 months of age. Numbers of analyzed fish are 11 (WT) and 12 (*notch3*^*fh334/*+^). **C** Percentage of alternations relative to all tetragrams at 17 months of age. Numbers of analyzed fish are 22 (WT) and 21 (*notch3*.^*fh334/*+^). **D** Total distance moved during the Y-maze test shown in (**C**). Error bars in **A**–**D** represent the mean ± SEM, with each dot indicating an individual value. n.s., not significant, *p < 0.05, ***p < 0.001, ****p < 0.0001. Data were analyzed using Tukey’s post hoc test following two-way ANOVA in **A** and **B** or unpaired two-tailed *t*-test in (**C**) and (**D**)

Cognitive decline is a major symptom that substantially reduces the quality of life in patients with CADASIL. Therefore, we next examined whether the *notch3*^*fh334/*+^ zebrafish recapitulate cognitive impairment. We performed an active avoidance test to assess the learning ability of individual zebrafish [90]. While learning ability remained intact in *notch3*^*fh334/*+^ zebrafish at 9 months of age, it was significantly reduced at 14 months of age (Fig. 2A and 2B). Importantly, this decline in learning ability occurred after the reduction in perfused cerebral vessel fraction, paralleling the sequence of pathological progression observed in human patients [75].

Additionally, we examined whether working memory is impaired in the *notch3*^*fh334/*+^ zebrafish using the Y-maze test. The percentage of alternations, an indicator of working memory, was significantly reduced in *notch3*^*fh334/*+^ zebrafish at 17 months of age (Fig. 2C). In contrast, the distance moved and total number of entries did not differ between WT and mutant zebrafish (Fig. 2D), suggesting that working memory is selectively impaired in *notch3*^*fh334/*+^ zebrafish.

These results indicate that cognitive function progressively declines with aging, consistent with observations in human patients.

### Aged *notch3*^*fh334/*+^ zebrafish exhibit abnormal protein aggregates around cerebral blood vessels

**Fig. 3:**
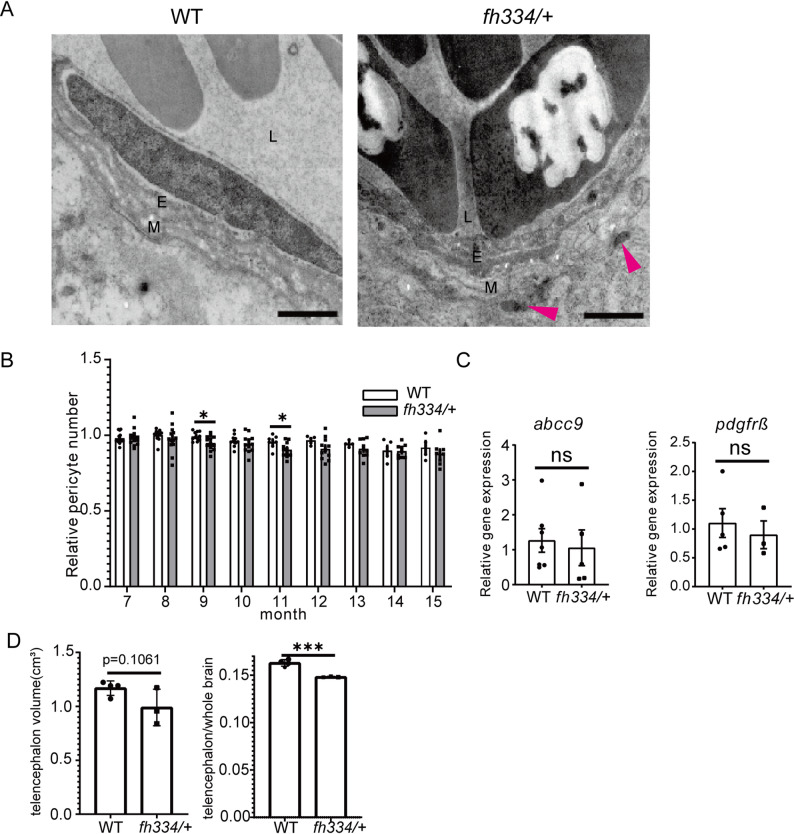
*notch3*^*fh334/*+^ zebrafish exhibit CADASIL-like histopathologies, including GOM deposits and brain atrophy **A** Representative electron microscopy images of cerebral blood vessels in *notch3*^*fh334/*+^ zebrafish at 22-24 months of age. Magenta arrowheads indicate osmiophilic deposits adjacent to mural cells. Numbers of analyzed vessels are 23 (WT) and 30 (*notch3*^*fh334/*+^). The number of analyzed fish is 4 per group. Scale bar = 1 µm. E, endothelial cells; M, mural cells; L, vessel lumen. **B** Longitudinal quantification of pericyte numbers in the same individuals at monthly intervals. Numbers of analyzed WT fish are 13 (7 months), 12 (8 months), 11 (9 months), 11 (10 months), 7 (11 months), 5 (12 months), 5 (13 months), 5 (14 months), and 4 (15 months). Numbers of analyzed *notch3*^*fh334/*+^ fish are 13 (7 months), 13 (8 months), 13 (9 months), 12 (10 months), 11 (11 months), 11 (12 months), 10 (13 months), 9 (14 months). **C** qRT-PCR analysis of *pdgfrß* and *abcc9* in whole brains of 18-month-old fish. Numbers of analyzed fish are 7 (WT) and 5 (*notch3*^*fh334/*+^) for *pdgfrß*, and 5 (WT) and 3 (*notch3*^*fh334/*+^) for *abcc9*. **D** Quantification of the whole brain volume in 16-month-old zebrafish by MRI analysis (left panel), and ratio of telencephalon volume to whole brain volume (right panel). Numbers of analyzed fish are 4 (WT) and 3 (*notch3*^*fh334/*+^). Error bars in **B**–**D** represent the mean ± SEM, with each dot indicating an individual value. *p < 0.05, ***p < 0.001. Data were analyzed using unpaired two-tailed *t*-test in (**B**–**D**).

We next examined whether the *notch3*^*fh334/*+^ zebrafish could recapitulate CADASIL pathologies at the histological and cellular level. One of the specific histopathological hallmarks of CADASIL is GOM deposits around mural cells, including pericytes and VSMCs [5]. GOM deposits are aggregated forms of abnormal proteins containing Notch3 and are used as a diagnostic marker for CADASIL [26, 30, 76]. We investigated whether GOM is present in our zebrafish model. Electron microscopy analysis of brains from *notch3*^*fh334/*+^ fish at 22–24 months of age revealed osmiophilic deposits around the vascular wall (Fig. 3A), indicating GOM accumulation in *notch3*^*fh334/*+^ zebrafish. These electron-dense deposits were detected in 33% (10/30) of the analyzed vessels in *notch3*^*fh334/*+^ zebrafish but were absent in WT controls at 22–24 months of age. This finding demonstrates that aged *notch3*^*fh334/*+^ zebrafish exhibit histopathological features mimicking the CADASIL hallmark, resembling features reported in mouse models [23, 61].

### Mural cell numbers are preserved in* notch3*^*fh334/*+^ zebrafish

In the cerebrovascular system, mural cells play a crucial role in maintaining blood vessel integrity and function [3, 71]. Mural cell loss and dysfunction have been reported in CADASIL patients and in CADASIL mouse models and are considered critical contributors to CADASIL pathology [14, 20, 39]. Therefore, we performed live-imaging analyses using *notch3*^*fh334/*+^ zebrafish crossed with *TgBAC(abcc9:gal4ff); Tg(UAS:EGFP); Tg(kdrl:DsRed2); nacre* zebrafish, in which pericytes and vascular endothelial cells are fluorescently labeled. We examined age-dependent changes in pericyte numbers within the same individuals. Although the number of pericytes in *notch3*^*fh334/*+^ zebrafish was significantly reduced at 9 and 11 months of age, the overall difference across the experimental period was relatively modest (Fig. 3B). Furthermore, the expression levels of *abcc9*, a marker of pericytes, and *pdgfrß*, a marker of both pericytes and VSMCs, remained unchanged at 18 months of age (Fig. 3C).

Collectively, these findings suggest that the CADASIL-like *notch3* mutation in *notch3*^*fh334/*+^ zebrafish does not lead to substantial pericyte loss prior to the decline in perfused cerebral vessel fraction or emergence of brain functional abnormalities.

### Aged notch3^*fh334/*+^ zebrafish exhibit brain atrophy

Brain atrophy is one of the key markers of CADASIL progression and is associated with cognitive decline, disability scales in cerebral microangiopathies, and lacunar lesions [17, 34, 67]. Moreover, white matter hyperintensity, a major pathological feature of CADASIL, contributes to the progression of brain atrophy [43]. Therefore, we analyzed brain volume in *notch3*^*fh334/*+^ zebrafish at 16 months of age using MRI. The telencephalon, a region homologous to human cerebrum, is known to be highly sensitive to ischemic injury in zebrafish compared with other regions of the brain [51, 93]. We therefore assessed whether the volume of the telencephalon was reduced in *notch3*^*fh334/*+^ zebrafish. Although the absolute telencephalic volume showed a downward trend in *notch3*^*fh334/*+^ zebrafish, the difference did not reach statistical significance, likely due to substantial inter-individual variability (Fig. 3D, left). As adult zebrafish are known to exhibit considerable variation in body size, typically ranging from 1.8 cm to 3.2 cm, even within the same age group [69], this variability may obscure localized tissue differences. To account for this variability, we evaluated the ratio of telencephalic volume to total brain volume as a more robust indicator of telencephalic atrophy. This normalized ratio was significantly lower in *notch3*^*fh334/*+^ zebrafish (Fig. 3D, right), indicating the presence of brain atrophy in this model. While our high-resolution MRI successfully detected telencephalic atrophy, focal lesions such as lacunar infarcts or white matter lesions were not observed (data not shown). This may reflect the inherent scale differences in cerebral small vessels: the diameter of cerebral small arteries in adult zebrafish is approximately 4–30 µm [86], whereas that of small penetrating arteries—the vessels implicated in human lacunar infarction – ranges from 40–900 µm [4].

### Integrated transcriptomic and proteomic analyses revealed reduced expression of type IV collagen-related molecules in *notch3*^*fh334/*+^ zebrafish

**Fig. 4:**
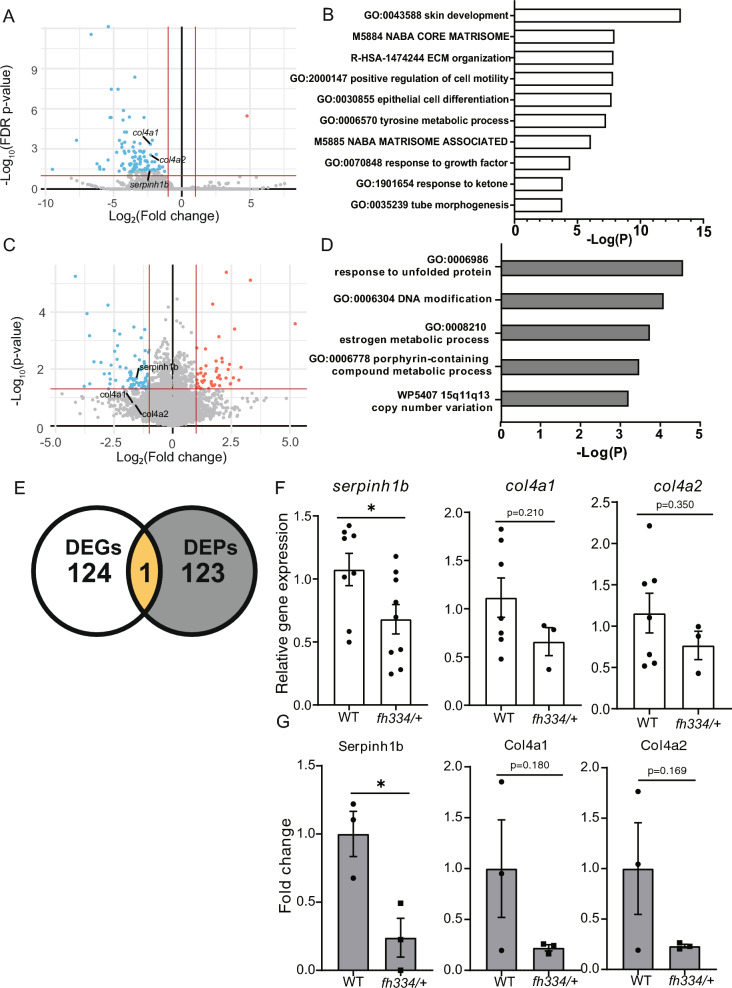
Multi-omics analysis identifies downregulation of type IV collagen-related molecules in aged *notch3*^*fh334/*+^ zebrafish brain. **A** Volcano plot of RNA-seq analysis in 18-month-old fish. DEGs extracted were defined by the FDR *p*-value < 0.1 and log_2_ fold change (FC) (abs, absolute value) > 0.5. The horizontal red line indicates FDR *p*-value < 0.1, and vertical red lines indicate log_2_ FC (abs) > 0.5. The number of analyzed fish is 3 per group. **B** Gene enrichment analysis of 71 human homologs among DEGs identified in the 3’RNA-seq analysis. **C** Volcano plot of LC–MS/MS proteomics analysis in 18- to 22-month-old fish. DEPs were defined by the *p*-value < 0.05 and log_2_ fold change (FC) (abs, absolute value) > 1. The horizontal red line indicates *p*-value < 0.05, and vertical red lines indicate log_2_ FC (abs) > 1. The number of analyzed fish is 3 per group. **D** Gene enrichment analysis of 85 human homologs corresponding to the DEPs identified in the LC–MS/MS analysis. **E** Venn diagram illustrating the overlap between the 125 DEGs and 124 DEPs. *serpinh1b* was identified as the sole common molecule between the two datasets. **F** Relative mRNA expression of *serpinh1b*, *col4a1* and *col4a2* analyzed by qRT-PCR using whole brain at 18-month-old *notch3*^*fh334/*+^ zebrafish brain. Numbers of analyzed fish are 8 (WT) and 9 (*notch3*^*fh334/*+^) for *serpinh1b*, 7 (WT) and 3 (*notch3*^*fh334/*+^) for *col4a1* and *col4a2*. **G** Fold changes in protein intensity for Serpinh1b, Col4a1, and Col4a2 based on LC–MS/MS data. The number of analyzed fish is 3 per group. Error bars in **F** and **G** represent the mean ± SEM, with each dot indicating an individual value. *p < 0.05, analyzed by unpaired two-tailed *t*-test

To investigate the mechanisms by which the Notch3 p.C680S mutation drives cerebrovascular and neurological abnormalities, we performed transcriptomic and proteomic analyses of aged *notch3*^*fh334/*+^ zebrafish. Transcriptomic profiling of 18-month-old *notch3*^*fh334/*+^ zebrafish identified 125 differentially expressed genes (DEGs) (up: 1 gene, down: 124 genes) (Fig. 4A; Supplementary Table S1, S2, S3). To enhance the functional interpretation of these results within the constraints of current zebrafish annotations, we performed gene enrichment analysis utilizing human orthologs. Of the 125 DEGs, 71 genes were successfully mapped to human orthologs and were subjected to functional enrichment analysis. Consequently, “skin development,” “NABA CORE MATRISOME”, and “extracellular matrix organization” emerged as the top three enriched terms (Fig. 4B). These results align with a previous study reporting that perturbations in the cerebrovascular matrisome and extracellular matrix play important roles in the pathological progression of CADASIL [32].

Parallel proteomic analysis of 18- to 22-month-old zebrafish identified 124 differentially expressed proteins (DEPs) (up: 46 proteins, down: 78 proteins) (Fig. 4C; Supplementary Table S4, S5, S6). Functional enrichment analysis was performed on 85 identifiable human homologs, revealing “response to unfolded protein,” “DNA modification” and “estrogen metabolic process” as the top three enrichment terms (Fig. 4D). Given that Notch3 mutations are associated with protein misfolding, these findings likely reflect a significant dysregulation of the unfolded protein response pathway in our model [66].

We then performed Principal Component Analysis (PCA) on normalized read counts from RNA-seq and normalized protein intensities from proteomics (Supplementary Fig. 3). For RNA-seq, PC1 and PC2 explained 77.8% and 11.2% of the variance, respectively. In contrast, for proteomics, they explained 42.6% and 21.5% of the variance. Both datasets demonstrated a clear separation between groups along the PC1 axis. The 95% confidence ellipses revealed within-group variability, which likely reflects expression changes influenced by environmental factors, particularly aging (Supplementary Fig. 3).

Integrated analysis of transcriptomic and proteomic data identified a single common factor Serpinh1b, overlapping between DEGs and DEPs (Fig. 4E). Serpinh1b is the zebrafish ortholog of human SERPINH1, a molecular chaperone specific to collagen [28]. Its expression level is closely associated with various types of collagens, including type IV collagen, whose subunit genes *col4a1* and *col4a2* were also among the DEGs identified in our transcriptomic analysis [28, 73, 91] (Fig. 4A). To validate these findings, we performed qRT-PCR analysis using whole brain samples from 18-month-old zebrafish. This confirmed a significant decrease in *serpinh1b* and downward trends for *col4a1* and *col4a2,* although the latter did not reach statistical significance (Fig. 4F). Consistent with these results, the proteomic data exhibited a trend toward reduced expression of Col4a1 and Col4a2 proteins (Fig. 4G).

To determine whether these molecular changes precede the onset of cognitive dysfunction or the reduction in cerebral perfusion, we quantified mRNA expression levels of *serpinh1b*, *col4a1,* and *col4a2* in 9- and 14-month-old zebrafish. qRT-PCR analysis of whole-brain samples revealed no significant changes in the expression of these three genes at either age (Supplementary Fig. 4). We further assessed the relationship between these transcript levels and learning performance. Notably, while no significant associations were observed at 9 months, 14-month-old *notch3*^*fh334/*+^ zebrafish exhibited a positive correlation between the expression of type IV collagen-related genes and learning performance (Supplementary Fig. 5).

Collectively, these results indicate that type IV collagen-related molecules are downregulated at both the transcriptomic and proteomic levels in *notch3*^*fh334/*+^ zebrafish in an age-dependent manner with their decline coinciding with or following the progression of cognitive impairment.

### Reduced perivascular Type IV collagen surrounding the cerebral blood vessels in aged *notch3*^*fh334/*+^ zebrafish

Type IV collagen is an important component of the cerebrovascular basement membrane (BM), and mutations in *col4a1* and *col4a2* are well-established causes of CSVD [42]. In murine models, loss-of-function or knockdown of *col4a1* or *col4a2* induces CSVD-like pathologies, primarily characterized by compromised BM integrity, impaired endothelial function, vascular wall thickening, and altered vascular stiffness [49]. Furthermore, a recent study demonstrated that *col4a1* deficiency in zebrafish also triggers CSVD hallmarks, including cerebral microbleeds and locomotor deficits [18].

**Fig. 5:**
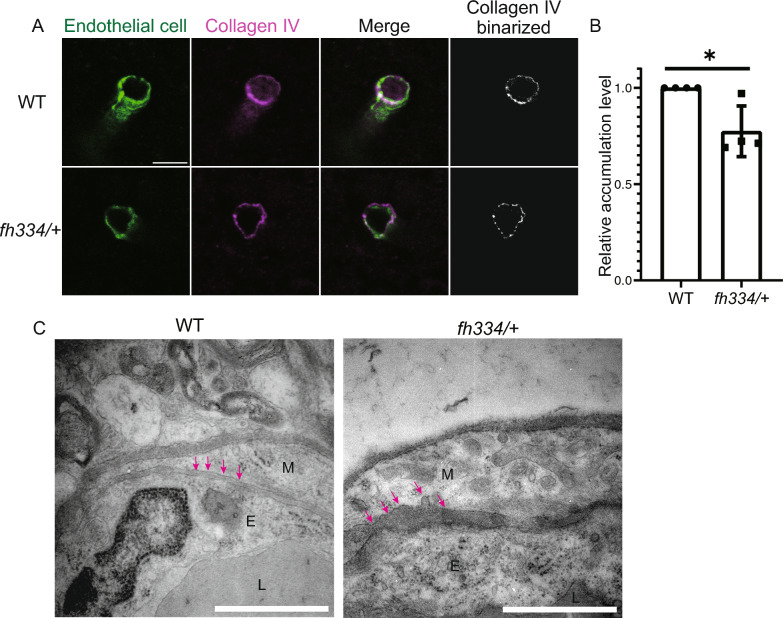
Reduction of perivascular type IV collagen and basement membrane disruption in aged *notch3*^*fh334/*+^ zebrafish. **A** Representative images of immunostaining of telencephalic blood vessels in 18-month-old *Tg(kdrl:EGFP)*^*s843*^*; notch3*^*fh334/*+^ zebrafish. EGFP-positive endothelial cells are shown in green, and type IV collagen in magenta. Binarized images of type IV collagen are shown in the right panel. Scale bar = 5 µm. **B** Quantification of type IV collagen-positive areas from the images in (**A**). A pair of zebrafish from each group was analyzed in each experiment. The collagen-positive area was normalized to the vascular perimeter. Relative accumulation levels were calculated by the mean of collagen-positive area in *notch3*^*fh334/*+^ zebrafish group relative to that in WT group in each experiment. At least 4 vessels were analyzed in each zebrafish. **C** Representative electron microscopy images of telencephalic blood vessels from *notch3*^*fh334/*+^ zebrafish at 22–24 months of age, obtained from the same experiment as Fig. 3A. Arrows indicate the subendothelial basement membrane. E, endothelial cells; M, mural cells; L, vessel lumen. Scale bar = 1 µm. Error bar in **B** represents the mean ± SEM, with each dot indicating an individual value. *p < 0.05, analyzed by paired *t*-test

Based on these observations, we hypothesized that the vascular dysfunction in *notch3*^*fh334/*+^ zebrafish stems from a reduction in type IV collagen. To test this, we examined the perivascular distribution of type IV collagen and confirmed a significant reduction in collagen-positive areas in *notch3*^*fh334/*+^ zebrafish aged over 18 months (Fig. 5A and 5B). Furthermore, ultrastructural analysis via electron microscopy revealed that the subendothelial BM in *notch3*^*fh334/*+^ zebrafish was uneven and exhibited inconsistent electron density compared to WT zebrafish at 22–24 months of age (Fig. 5C). These morphological abnormalities—indicative of BM disruption—align with the phenotypes reported in *col4a1* loss-of-function mouse models [22]. Such defects were observed in 7 out of 17 vessels with clearly discernible BMs in *notch3*^*fh334/*+^ zebrafish, whereas only 1 out of 12 such vessels showed similar features in WT controls. These data suggest that the diminished type IV collagen surrounding the cerebral vasculature contributes to the pathogenesis of CADASIL.

### The structure of the mutant Notch3 protein might be important for the collagen accumulation trait

**Fig. 6:**
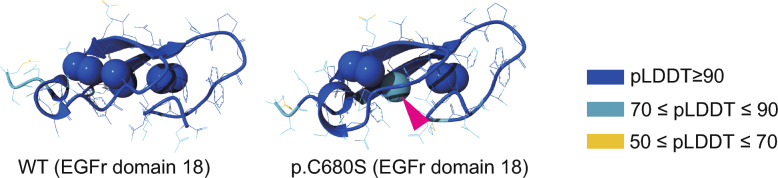
Structural modeling classifies the *notch3*^*fh334/*+^ zebrafish variant as a nonconventional mutation. Predicted structural models of the zebrafish Notch3 EGFr18 domain generated by AlphaFold 3. Models are colored according to the predicted local distance difference test (pLDDT) scores. Sulfur atoms of cysteine residues are represented as spheres. The p.C680S mutation results in an unpaired cysteine residue (arrowhead) positioned at the center of 3 pairs of disulfide bonds, without any apparent solvent-exposed cysteine residues

A reduction in type IV collagen was previously reported in a CADASIL patient harboring the cysteine-sparing p.R213K mutation in NOTCH3 [57, 72]. Recently, NOTCH3 mutations were classified into “conventional” and “nonconventional” types based on structural and pathophysiological characteristics [27]. Nonconventional mutations are associated with a pro-hemorrhagic CADASIL phenotype and typically involve central cysteine residues without creating an apparent solvent-exposed cysteine [27]. Our structural analysis using AlphaFold 3 indicated that the NOTCH3 p.R213K mutation lacks an evident solvent-exposed cysteine (Supplementary Fig. 6), suggesting that it may also fall into the nonconventional category. In contrast, type IV collagen accumulation in the vascular media has been reported in patients with major conventional NOTCH3 mutations [12]. Collectively, these observations raise the possibility that reduced type IV collagen is a distinctive pathological feature specifically associated with nonconventional NOTCH3 mutations. Consequently, we hypothesized that the p.C680S mutation in our *notch3*^*fh334/*+^ zebrafish represents a nonconventional Notch3 variant. AlphaFold 3-based structural modeling of the zebrafish Notch3 EGFr18 domain confirmed that the p.C680S mutation does not result in evident solvent-exposed cysteine residues (Fig. 6). These findings support the classification of the p.C680S variant as a nonconventional mutation, which likely underlies the pro-hemorrhagic phenotype observed in our zebrafish model.

## Discussion

In this study, we established a zebrafish model carrying the Notch3 p.C680S mutation, which successfully recapitulates key age-related features of CADASIL, including cerebrovascular dysfunction, cognitive impairment, and granular osmiophilic material (GOM). Our findings suggest that age-dependent cognitive decline in *notch3*^*fh334/*+^ zebrafish is driven by the chronic hypoperfusion and subsequent brain atrophy. These results underscore the utility of our zebrafish CADASIL model as a complementary model for investigating CADASIL pathogenesis, particularly regarding nonconventional Notch3 mutations. While various CADASIL mouse models have been developed, they often fail to fully mirror human pathology. A transgenic mouse model carrying human NOTCH3 p.R182C showed GOM around blood vessels but did not show white matter lesions [23, 64]. Murine models carrying rat NOTCH3 p.R169C or human NOTCH3 p.R90C mutations have been developed [13, 33, 61], and in some cases, mild cognitive deficits have been reported [15, 46]. Even in knock-in (KI) mouse models (p.R170C or p.R142C), which avoid overexpression artifacts, they often show incomplete phenotypes [47, 81]. The KI model with p.R170C displayed several CADASIL symptoms, including GOM deposits and white matter lesions, but lacked VSMC abnormalities and cognitive impairment [81]. Surprisingly, the KI model with p.R142C did not develop any CADASIL-like phenotypes, including GOM deposits and arteriopathy [47]. In contrast, our study demonstrated that *notch3*^*fh334/*+^ zebrafish, carrying p.C680S mutation, faithfully reproduce major pathological features of human CADASIL, such as vascular dysfunction and cognitive impairment in a temporal sequence that closely mirrors clinical progression in human patients. This may be attributed to the fact that zebrafish aging—specifically the brain clearance system and lifespan—more closely resembles human aging than murine models do, making zebrafish a highly relevant platform for studying age-related Notch3 pathologies [35].

We also provide new insights into the role of type IV collagen-related genes, specifically *serpinh1b*, *col4a1,* and *col4a2*, which were downregulated in our model. Previous clinical data indicate that type IV collagen reduction is associated with nonconventional mutations, but not with conventional mutations [12, 57, 72]. Given that *col4a1* and *col4a2* mutations are established causes of CSVD, their reduced expression likely leads to the compromise of the cerebrovascular basement membrane (BM), resulting in intracerebral microbleeds and abnormal vasoconstriction [44, 49, 89]. Our model’s BM disruption parallels that of *col4a1* mutant mice, strengthening the link between collagen deficiency and the CADASIL vascular phenotype in the context of nonconventional mutations [22]. Although transgenic mice carrying the nonconventional mutation (p.R75Q) exhibit granular osmiophilic material (GOM) accumulation, no apparent brain abnormalities have been reported [21]. Consequently, investigating nonconventional Notch3 mutants in zebrafish is of particular importance. Nonetheless, this remains a preliminary interpretation that warrants further validation through comparative analyses across diverse NOTCH3 mutation types and the examination of human clinical samples.

The precise mechanism underlying reduced type IV collagen levels in aged *notch3*^*fh334/*+^ zebrafish remains to be fully elucidated, but our data highlight Serpinh1b (the zebrafish homolog of SERPINH1/HSP47) as a key mediator. Given that the *Notch3* mutations associated with CADASIL do not generally compromise canonical Notch3 signaling [9], non-canonical mechanisms likely underlie the reduction in type IV collagen. Notably, a positive correlation between SERPINH1 (the human homolog of zebrafish Serpinh1b) and type IV collagen has been established in neuroblastoma cells [91]. Moreover, *serpinh1* knockout mice embryos display discontinuous BM due to impaired type IV collagen secretion [48]. These findings suggest that the reduction in *serpinh1b* may play a critical role in decreasing type IV collagen and causing subsequent BM disruption, ultimately leading to the vascular dysfunction observed in our model. Age-related factors likely drive the regulatory mechanisms underlying *serpinh1b* downregulation. *serpinh1* expression is known to decrease with age in the murine brain, kidney, and pancreas [62]. Recent evidence further indicates that impaired glymphatic function in a CADASIL mouse model correlates with cellular senescence within the neurovascular unit, including neurons and microglia [45]. While perivascular cells were not specifically addressed in that study [45], it is plausible that aging, exacerbated by glymphatic dysfunction in the context of NOTCH3 mutations, leads to the downregulation of *serpinh1b* expression. This regulatory link suggests that reduced *serpinh1b* expression could directly contribute to the diminished type IV collagen levels observed in the zebrafish model.

In conclusion, this is the first study to establish a zebrafish model of CADASIL. Our findings suggest a novel pathogenic mechanism involving *serpinh1b*-mediated collagen deficiency and emphasize the importance of genotype–phenotype classification. While questions remain regarding the exact mechanisms of reduced vessel perfusion and Notch3 protein accumulation, the zebrafish offers distinct advantages for future research, including its suitability for live imaging and its physiological similarity to human aging. Expanding this model to include diverse NOTCH3 mutations will be instrumental in identifying new therapeutic targets for CADASIL.

## Supplementary Information

Supplementary Material 1.

Supplementary Material 2.

## Data Availability

The datasets supporting the conclusions of this article are included within the article and its additional files.

## Abbreviations

CADASIL: Cerebral autosomal dominant arteriopathy with subcortical infarct and leukoencephalopathy
CSVD: Cerebral small vessel disease
GOM: Granular osmiophilic material
VSMC: Vascular smooth muscle cell
CBF: Cerebral blood flow
DEG: Differentially expressed gene
CPM: Counts per million mapped reads
DEP: Differentially expressed protein
BM: Basement membrane
KI: Knock-in
