# Supplementary material for: Age-dependent vascular and neurological characteristics of CADASIL are recapitulated in Notch3 mutant zebrafish, implicating a role for type IV collagen in disease progression
Source: Acta Neuropathol Commun. 2026 Jun 3;14:163. doi: 10.1186/s40478-026-02333-8 (PMC13455480; doi:10.1186/s40478-026-02333-8)
Supplement: Supplementary file 1 [file 40478_2026_2333_MOESM1_ESM.pdf]

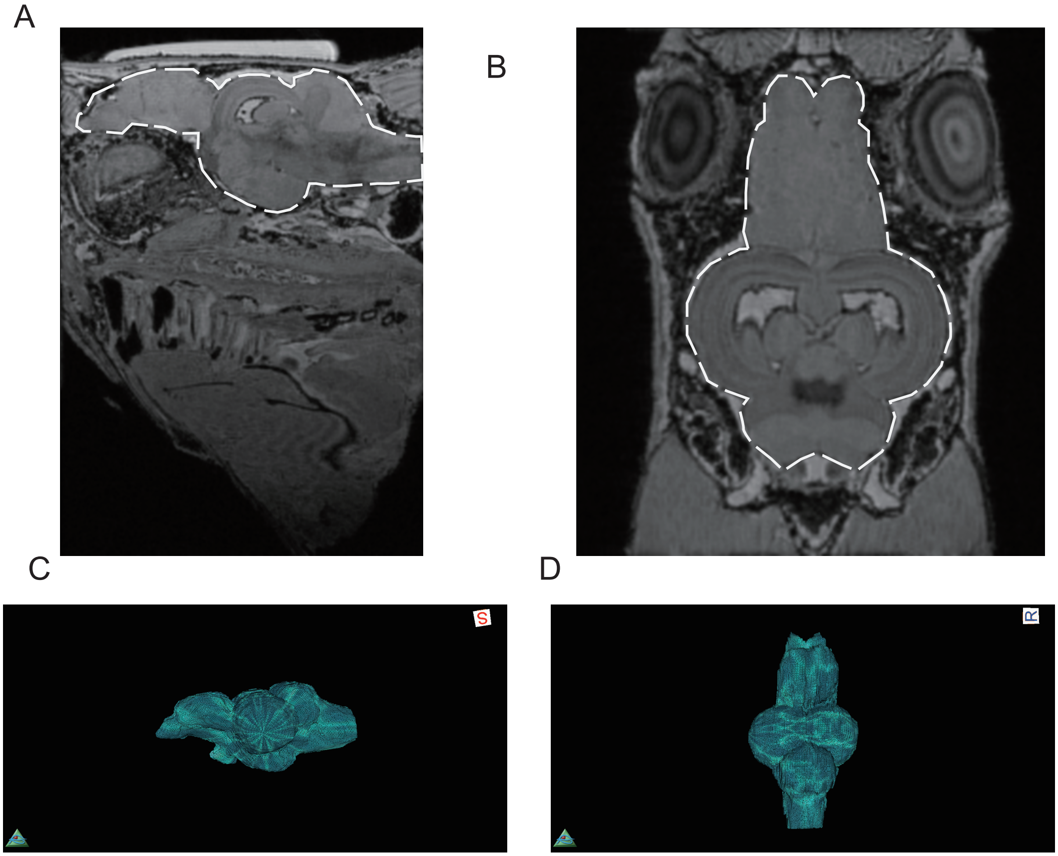

Supplementary Fig. 1 Representative MRI analysis and brain volume measurement.

(A, B) Representative MRI slices of the zebrafish brain in lateral (A) and dorsal (B) views. White dotted line indicates the outline of the whole brain.

(C, D) Representative 3D modeling and volume analysis of the whole brain based on MRI data. The analysis was conducted using OsiriX software.

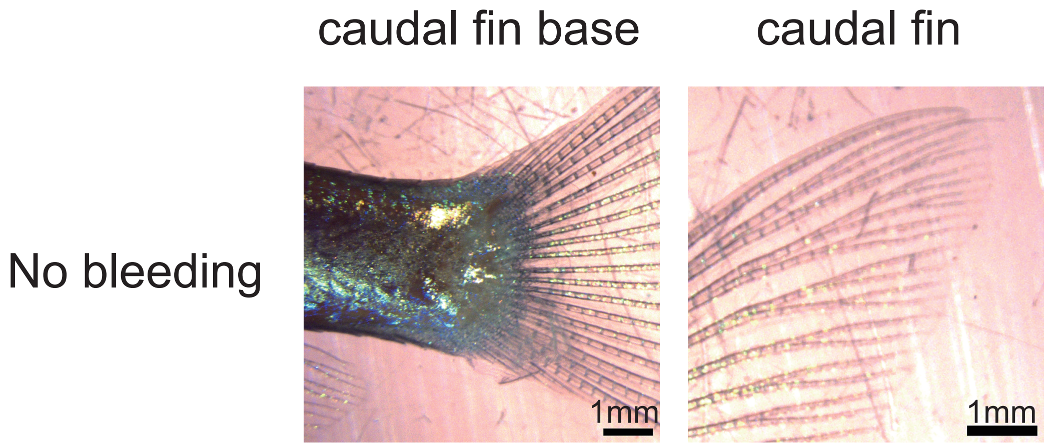

Supplementary Fig. 2 Individual without bleeding associated with Fig. 1C. Scale bar = 1mm.

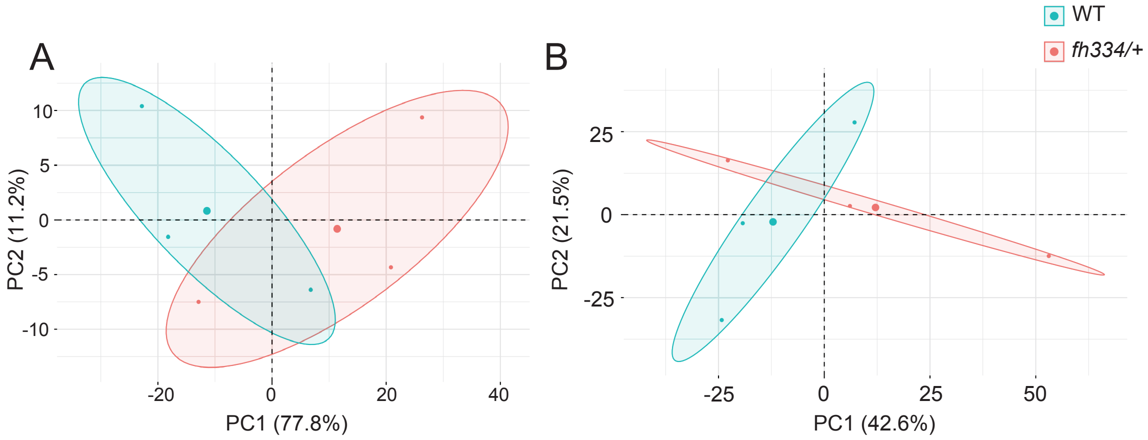

Supplementary Fig. 3 PCA based on transcriptome and proteome associated with Fig. 4 PCA based on transcriptome (A) and proteome (B). Each point represents an individual sample, and large points indicate the centroid of each group. Ellipses represent the 95% confidence interval around the group centroids.

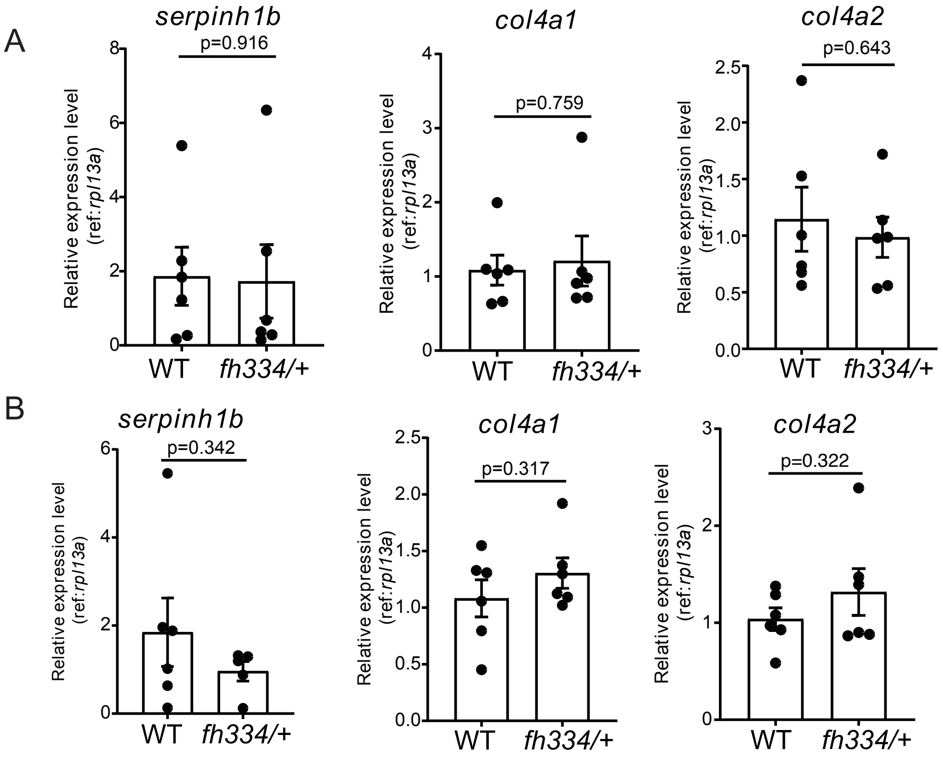

Supplementary Fig. 4 mRNA expression levels of type IV collagen-related genes are not changed at 14 months of age and 9 months of age *notch3*$^{fh334/+}$ zebrafish.

(A) The fold change of mRNA expression levels of serpinh1b, col4a1 and col4a2 at 14 months of age. Number of analyzed fish are 6 (each group)

(B) The fold change of mRNA expression levels of serpinh1b, col4a1 and col4a2 at 9 months of age. Number of analyzed fish are 6 (WT) and 5 (notch3fh334/+) in serpinh1b, 6 (each group) in col4a1 and col4a2.

Error bars are represented as mean ± SEM. P-value was analyzed by unpaired two-tailed t-test.

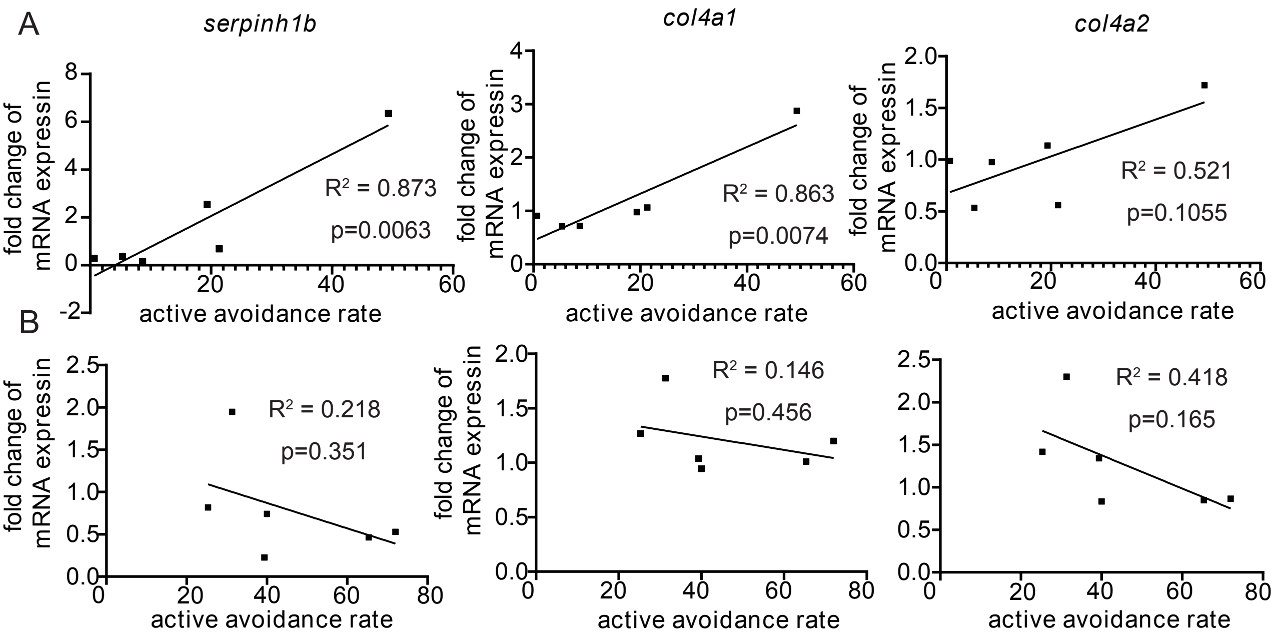

Supplementary Fig. 5 Learning ability and mRNA expression level are positively correlated at 14 months of age *notch3*$^{fh334/+}$ zebrafish

These graphs show the relationship between the mRNA expression level of type IV collagen-related genes and the active avoidance rate at 14 months of age (A) and 9 months of age (B). Each dots represents an individual value of *notch3*$^{fh334/+}$ zebrafish. Each black line indicates a regression line generated by simple linear regression analysis with Graph Pad Prism.

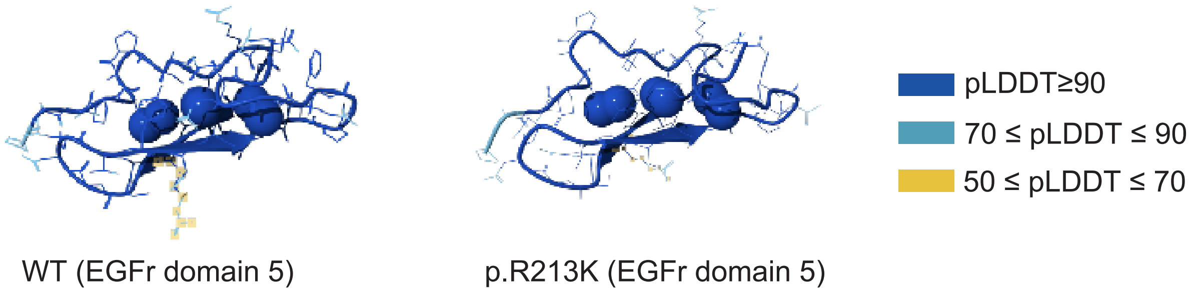

Supplementary Fig. 6. The p.R213K mutation of human Notch3 do not have clear solvent exposed cysteine.

The structural analysis of human Notch3 EGFr5 domain predicted by Alpha fold 3. The predicted structures were colored according to the predicted local distance difference test (pLDDT). The sulfur atoms of the cysteine residues are represented by spheres. The square dots in the structure represents the atomic positions of 213rd amino acid.
